# The Influence of Selected Factors on the Aqueous Cryptotanshinone Solubility

**DOI:** 10.3390/pharmaceutics13070992

**Published:** 2021-06-30

**Authors:** Justyna Kobryń, Jowita Dałek, Witold Musiał

**Affiliations:** Department of Physical Chemistry and Biophysics, Faculty of Pharmacy, Wroclaw Medical University, ul. Borowska 211A, 50-556 Wroclaw, Poland; justyna.kobryn@umed.wroc.pl (J.K.); jowita.kaluzna@gmail.com (J.D.)

**Keywords:** cryptotanshinone, solubility, alcoholamines, HPLC

## Abstract

The application of cryptotanshinone (CT), a diterpenoid obtained from the root of *Salviae miltiorrhiza*, is significantly hindered due to its poor aqueous solubility. The aim of the present research was to develop an optimal solvent for analytical and preparative procedures of prospective dermal hydrogel formulations with CT. The influence of pH, temperature, and cosolvent presence on the solubility of CT was examined. Various components were applied to increase CT solubility, i.e., ethanol, 2-amino-2-methyl-1,3-propanediol, 2-amino-2-(hydroxymethyl)-1,3-propanediol, 2,2′,2″-nitrilotriethanol, and triisopropanoloamine. The concentration of CT was analyzed by spectral and chromatographic methods, including UV–vis and HPLC methods. The increased solubility of CT was demonstrated in alkaline solvents with ethanol as a cosolvent. CT solutions doped with alcoholamines are more stable compared to CT solutions doped with NaOH.

## 1. Introduction

Solubility is an important factor determining the pharmaceutical availability and bioavailability of a drug. It influences the optimal form of the preparation administered to the patient and has a significant effect on the blood concentration of the drug and the expected pharmacological response [1]. Drugs of weak aqueous solubility, presumed for circulatory absorption, often require higher doses than water-soluble medicines. More than 40% of newly developed pharmaceuticals have very low solubility; therefore, the problem is significant in the context of formulation and pharmacokinetics [2]. Many biologically active substances of natural origin are characterized by low water solubility. One of them is cryptotanshinone (CT) [3].

The compound has been isolated from *Salviae miltiorrhizae radix et rhizoma* (*Lamiaceae*) and is widely studied [4,5,6,7,8,9,10]. It was proposed for the prevention and therapy of cardiovascular diseases [11], cognitive disorders [12], kidney and liver dysfunctions [13,14], and gynecological diseases [15]. In vitro and in vivo studies in rats presented reduced oxygen consumption in the myocardium and improved coronary circulation [16], as well as cardioprotective [17,18] and hypotensive activity [19]. Neuroprotective properties in Alzheimer’s disease [20], as well as anticancer properties, were reported [4,6,21,22,23]. Antimicrobial activity in a wide range of infections by Gram-positive and Gram-negative bacteria, as well as other microorganisms, was evaluated [6,10,11,24,25,26].

**Figure 1:**
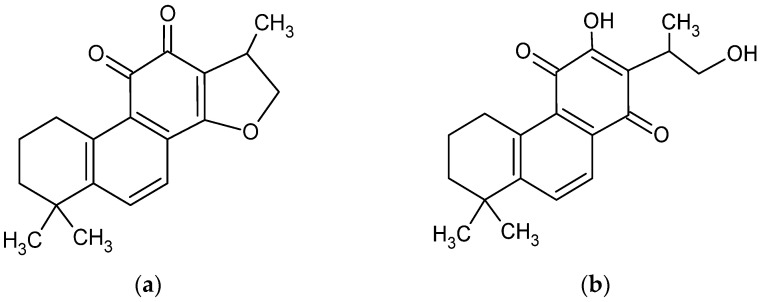
Chemical structure of cryptotanshinone (1,2,6,7,8,9-hexahydro-1,6,6-trimethyl-(R)-fenanth [1,2-b] furan-10,11-dione) (**a**) [4] and its enol form (**b**) [28].

CT is a diterpene compound (Figure 1) of 4.9 pKa. CT. As a lipophilic compound, it is slightly soluble in water (0.00976 mg/mL) [3] but very soluble in dimethyl sulfoxide, methanol, ethanol, chloroform, and ether [27].

In the last two decades, the dissolution and bioavailability of CT has been studied, including very modern approaches, such as solid dispersion [14,27,29,30], ethosomal [31], niosomal [32], cerasomal [33], and solid lipid nanoparticles [30], and cyclodextrin complexation [22,34]. Studies of the influence of pH on CT solubility revealed structural modifications of the compound above pH 11.0 and even destruction below pH 1.0, which was discovered during research on fluorescence properties [35], whereas the highest stability of CT structure was recorded between pH 8.0 and pH 12.0 in absorbance tests using the UV–vis method [34]. The tetrahydrofuran ring of CT collapsed in strongly alkaline conditions [28,36] with an enol form of tanshinone V has been established (Figure 1b).

**Figure 2:**
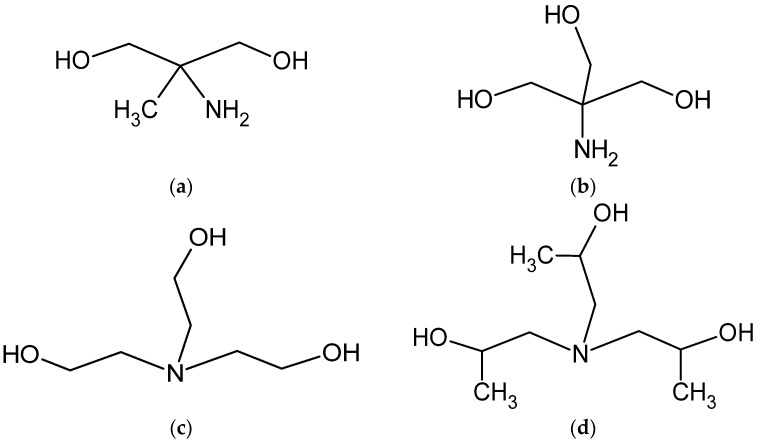
Schematic representation of the variability of the applied alcoholamine molecules: AMPD (**a**), TRIS (**b**), TEOA (**c**), and TIPA (**d**).

The alkaline environments may be induced both by strong inorganic hydroxides, e.g., NaOH, or by alcoholamines, which are bifunctional components, combining the properties of alcohols and amines [37]. Aminomethyl propanediol (AMPD), tromethamine (TRIS), triethanolamine (TEOA), and triisopropanolamine (TIPA) are components of high interest due to potential application in pharmaceutical and cosmetic industries as fatty acid binders [37,38,39] (Figure 2). TEOA is applied as antiacne preparation in the form of salicylate. AMPD may react with the methyl esters of the organic acids forming amides [40]. TRIS and TIPA may be used in the synthesis of surface-active agents as emulsifying agents and emulsion stabilizers in pharmaceutical applications [41,42]. TIPA reacts rapidly with acids forming amine salts [41]. AMPD and TIPA enable pH neutralization in cosmetic preparations, e.g., lotions, aerosols, and tonics [40,41]. TRIS is administered for the treatment of salicylates, barbiturates, and methanol intoxications, and it may be used for plasma alkalization [42]. NaOH and alcoholamines are used in pharmaceuticals and could serve as potential ingredients of the preparation for external application. Application of selected alkaline components may enhance the aqueous solubility of CT in pharmaceutical preparation.

The aim of this work was to evaluate the aqueous solubility of CT in an alkaline environment and the selected factors influencing the process of dissolution.

## 2. Materials and Methods

### 2.1. Materials

Cryptotanshinone of 99.26% purity (Selleckchem, Poznań, Poland), ≥99.9% methanol for HPLC (Chempur, Piekary Śląskie, Poland), 96% ethanol (Stanlab Sp. J., Lublin, Poland), ≥99.5% isopropanol (Fluka: BioChemika, Buchs, Switzerland), sodium hydroxide (Stanlab Sp. J.), 2-amino-2-methyl-1,3-propanediol (AMPD, Sigma-Aldrich, Poznań, Poland), 2-amino-2-(hydroxymethyl)-1,3-propanediol (TRIS, Sigma-Aldrich), 2,2′,2″-nitrilotriethanol (TEOA, Sigma-Aldrich), tris(2-propanol)amine (TIPA, Sigma-Aldrich), 96% sulfuric acid (VI) (Stanlab Sp. J., Lublin, Poland), 35–38% hydrochloric acid (HCl, POCH BASIC, Gliwice, Poland), acetonitrile for HPLC (Sigma-Aldrich), formic acid for HPLC (Sigma-Aldrich), and deionized water were used in the experimental procedures.

### 2.2. Preliminary Evaluation of the CT Solubility Performance in Various Solvents

The visual appearance of the CT samples in a variety of solvents was evaluated. The solvents presented in Table S1 (Supplementary Materials) were used. A 1 mg amount of CT was weighed and placed in 2 mL of solvent in a test tube, as described in Table S1 (Supplementary Materials). Observations were made after 24 h and after 8 days. The tested samples were protected from light at 25 °C.

For microscopic evaluation, samples of 0.2 mg/mL CT concentration in 96% ethanol (S1), aqueous solution of 8% ethanol (S2), and aqueous solution of 8% ethanol with a 0.1 mol/L solution of NaOH were prepared (S3) and assessed with an optical microscope (Delta Optical Evolution 100) with photo processing software (DLT-Cam PRO 18MP, Delta Optical, Poland).

The influence of temperature and sonification was further evaluated on the selected solvent: 0.1 mol/L NaOH. The system of 5 mg of CT and 100 mL of 0.1 mol/L NaOH was sonicated at 40 kHz (Sonic-5, Polsonic, Warsaw, Poland) for 1, 5, or 10 min at temperatures of 30 ± 1.5, 40 ± 1.5, 50 ± 1.5, and 60 ± 1.5 °C. The resulting change in color was recorded as present or absent.

### 2.3. Absorbance Studies of CT in Varied Alkaline Solvents

A standard curve for CT was prepared in methanol at a wavelength of 271 nm [19], according to available literature [43]. The achieved standard curve prepared by the methanol solution equation was y = 0.052x, and the linear determination index R^2^ was 0.9998 (*n* = 3). The UV–vis spectra of CT in Samples 2 and 8–11 were evaluated using a UV–vis spectrophotometer (Halo DB-20, Dynamica, Poland) in the range of 245–300 nm. The determination of the content of alkaline groups that could potentially bind acidic groups of cryptotanshinone was based on the modified pharmacopoeial method for the acid number determination [44]. In the modified method, the absorbance of Samples 2 and 8–11 after sonication for 10 min in 60 ± 1.5 °C was measured at 271 nm. Solvent 2 or 8–11 was added in 1 mL portions until a volume of 8 mL was obtained, and the absorbance was measured after every 1 mL.

### 2.4. Potentiometric and Conductometric Studies

Samples P1–P2 presented in Table S2 (Supplementary Materials) were titrated with 0.1 mL portions of 0.001 mol/L NaOH until a volume of 10.0 mL was obtained in a TitraLab AT 1000 Series titrator potentiometer (Hach Lange, Düsseldorf, Germany), with a CC-505 conductivity sensor (Elmetron, Zabrze, Poland) and a PHC 805 pH-electrode (Hach Lange, Düsseldorf, Germany), to provide an insight into the initial phase of CT neutralization. In P2, 1.2 mL of 96% ethanol as a cosolvent was added to 5.0 mg of CT and left for 20 h, then 35 mL of distilled water was provided. The solutions without CT, P1r and P2r, were used as blank samples.

### 2.5. HPLC Analysis

The HPLC method was used to overcome the limitations of the UV–vis spectrophotometric method due to the standard curve. The analytical methods of tanshen and tanshinones were previously been performed [5,8,43]. The method was modified to analyze the concentration of CT in the studied solutions. The method was set as follows: a flow rate of 0.4 mL/min, a column temperature of 40 °C, and a wavelength of 280 nm were applied. The mobile phase consisted of distilled water, 0.1% formic acid (phase A) and acetonitrile, and 0.1% formic acid (phase B). The gradiented A/B was set, respectively, as 98:2–1:99 and 1:99–98:2 *v*/*v*% for 28 min. Powdered CT of purity 99.26%, dissolved in methanol, was used as a standard. To adapt to the concentration range, two standard curves were prepared: one for alcoholamine solutions (S1) and the other for the NaOH solutions (S2).

The HPLC system (Ultimate 3000 model, Thermo Fisher Scientific, Schwerte, Germany) consisted of a pump (LPG-3400SD), an autosampler (WPS-3000TSL), a column thermostat (TCC-3000SD), a UV detector (UV, DAD-3000), and a column (Kinetex 2.6 µm C18 100Å, Phenomenex, Torrance, CA, USA). The solutions were filtered through 0.22 µm pores filters. The mixtures were prepared using an ultrasonic washer for 10 min at 40 ± 1.5, 50 ± 1.5, and 60 ± 1.5 °C and ethanol as a cosolvent at an amount of 8% *w*/*w*. Various concentrations of NaOH and different temperatures of 1% alcoholamine exposure to ultrasound waves were tested (Table S3, Supplementary Materials). The chromatographic measurements of CT in ethanol (E) and water (W) were executed as standards. The alkaline solvents were added to obtain a saturated solution of CT (approximately 1.0 mg of CT in 5.0 g of solvents). HPLC measurements were performed after preparation and after 1, 2, 3, 6, and 8 weeks. The pH measurements of the samples designed for HPLC assays were carried out on the first day of sample preparation at a temperature of 25.0 ± 0.5 °C.

## 3. Results

### 3.1. Visual Appearance of CT–Solvent Systems

**Figure 3:**
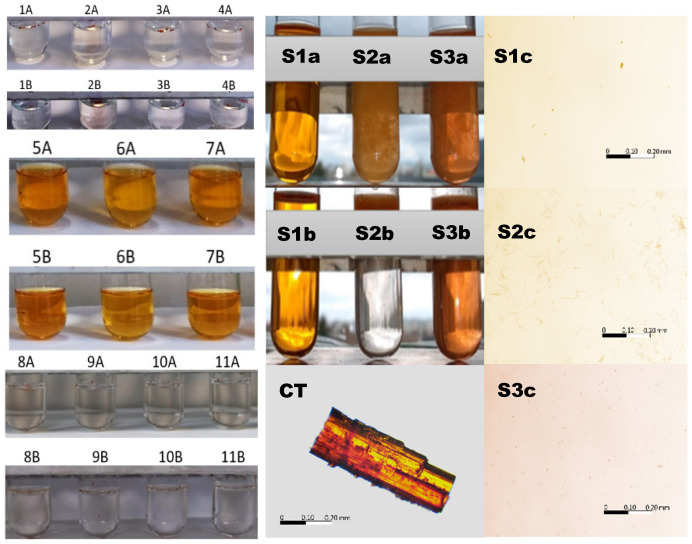
Left panel: macroscopic illustration of CT in various solvents and color intensity on the first (**A**) and eighth days (**B**) after dissolution in the solvents: distilled water (**1**), 0.1 mol/L sodium hydroxide (**2**), 1.0 mol/L sulfuric acid (VI) (**3**), 0.5087 mol/L hydrochloric acid (**4**), methanol (**5**), ethanol (**6**), isopropanol (**7**), AMPD (**8**), TRIS (**9**), TEOA (**10**), and TIPA (**11**). Middle panel: macroscopic illustration of CT in 96% ethanol (**S1**), 8% ethanol (**S2**), and 8% ethanol with a 0.1 mol/L solution of NaOH (**S3**). Illustration of the influence of ethanol on the solubility of CT in the 0.1 mol/L solution of NaOH immediately (**a**) and after 5 min (**b**), and a microscope image of the powdered CT particle in the air. **Right panel**: microscope images of a drop taken from the surface of the samples in the test tubes S1b, S2b and S3b, respectively. The bar represents the same longitude (200 µm). The details are in the text.

The color intensity in the test tubes after 24 h and after 8 days in the respective solvents doped with CT is presented in Figure 3 (left panel). The most intense color was observed in the case of methanol, ethanol, and isopropanol, which was derived from dissolved CT. The aqueous samples, acidic samples, and alcoholamine samples remained colorless with undissolved particles of CT floating on the surface of the solutions. The exception was the NaOH solution, where, in addition to floating particles of CT, slight color was observed.

The 96% ethanolic solutions were transparent and colorful directly after the implementation of CT and after 5 min (S1a, S1b), with separated particles of undissolved CT in the observation field (S1c), whereas the CT aqueous dispersion doped with 0.5 mL of ethanol (8% ethanol) provided a turbid system, with observable, floating, insoluble CT particles (S2a, S2b) of longitude in the range of 25 µm (S2c). The solution was colorless. The system with a low ethanol content (8%), but with a 0.1 mol/L solution of NaOH, showed turbidity; however, the entire solution was colored, which indicated the partial dissolution of CT. The solid particles also floated, and their dimensions were in the range of 5 µm (Figure 3, right panel). 

A further evaluation of CT in the presence of 0.1 mol/L NaOH revealed color in the selected samples, whereas the others were colorless, as presented in Table 1. For the summarized factors overcoming a time of 5 min and a temperature of 40 °C, the color appeared what was accepted as CT solubilization in the respective fluid.

### 3.2. UV–Vis Spectrophotometric Evaluation of CT Solubility

**Figure 4:**
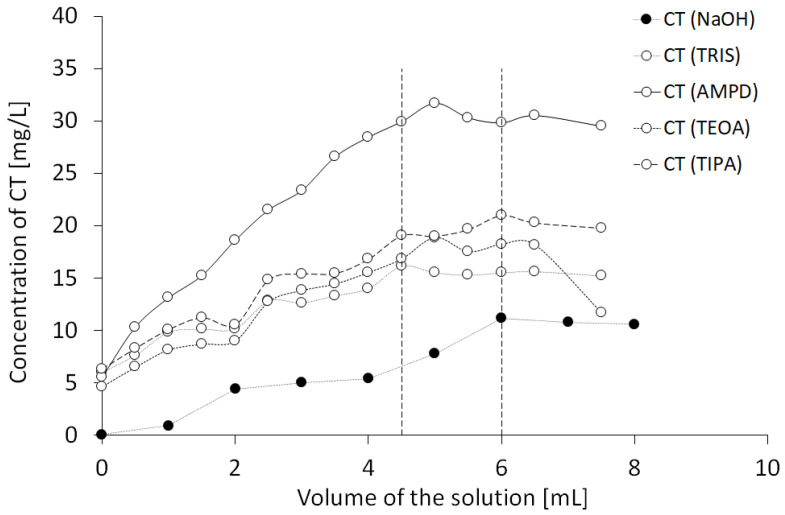
Influence of solutions (0.001 mol/L NaOH, 1% AMPD, 1% TRIS, 1% TEOA, and 1% TIPA (n = 3)) on the spectrophotometrically estimated neutralization of CT by the alkaline components.

In order to evaluate the neutralization of acidic groups of CT via alkaline components, the CT solution was combined with increasing volumes of NaOH solution or alcoholamine solutions, as presented in Figure 4. The equilibrium was observed at 6 mL of 0.001 mol/L NaOH added to 5 mg of CT, whereas the equilibrium point for alcoholamines was in the range of 4.5–6 mL.

The resulting molar proportion of NaOH/CT was ca. 27, whereas the same amounts of CT solved in various alcoholamine solutions provided varied proportions (Table 2). The equilibrium state in the case of the AMPD solution as a solvent for CT provided a molar ratio of AMPD/CT 889, and for the TRIS solution, the molar ratio of TRIS/CT in the equilibrium state was 1514. Similar ratios were observed, as when TEOA was applied, the TEOA/CT molar ratio reached a value of 1089. The lowest molar ratio was recorded in the TIPA solution, where the value was 737 in the equilibrium state. The mean pH values of the overtitrated systems at 8 mL are presented in Table 2 and were 10.07, 10.08, 9.16, 9.46, and 9.18.

### 3.3. Titration Studies

**Figure 5:**
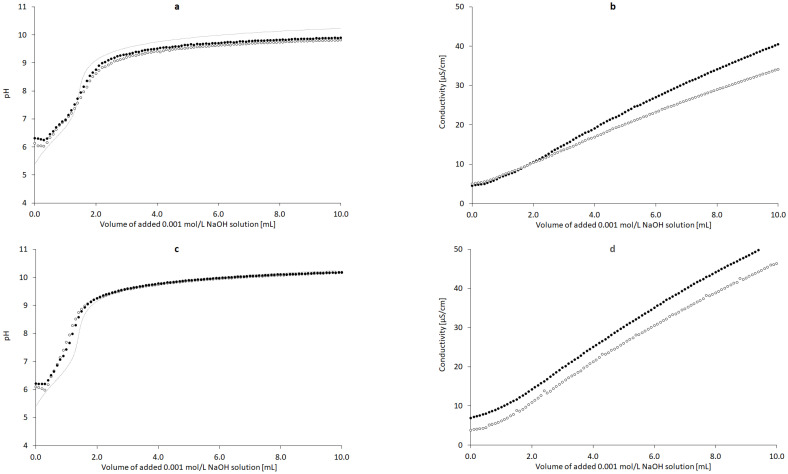
Influence of added volume of the 0.001 mol/L NaOH solution on the pH and conductivity of the dispersion of 5 mg of nondoped CT ((**a**,**b**), *n* = 3) and CT doped with 3.3% ethanol *w*/*w* ((**c**,**d**), *n* = 3); deionized water (◦), deionized water with CT (•), theoretical pH plot presenting the influence of ca. 0.004 mmol/L CO_2_ from the atmosphere (^…^).

The addition of the 0.001 mol/L solution of NaOH resulted in a respective increase in pH and conductivity (Figure 5a,b). The range of the pH of CT in water titrated by 0.001 mol/L NaOH was 6.37–10.09, and a plateau phase was established. In the case of conductivity, the range was 0.00045–0.00516 S/m. The range of the pH of CT in 3.3% ethanolic solution titration with 0.001 mol/L NaOH was 6.21–10.18, and a plateau phase was established, as in the previous case (Figure 5c). The range of conductivity was 0.00069–0.00663 S/m (Figure 5d). Theoretical potentiometric titration plot was added to Figure 5a,c to reflect the influence of CO_2_ from the atmosphere in a concentration of ca. 0.004 mmol/L, which could interfere with the results.

### 3.4. HPLC Studies

Equations of standard plots for the evaluation of CT concentration in the HPLC assays are presented in Table 3.

**Figure 6:**
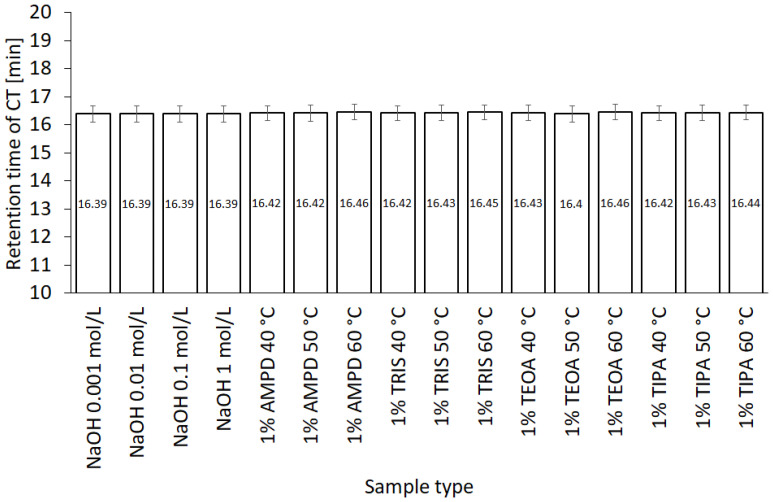
Variability of CT retention times in various solvents (*n* = 3).

The quantification of CT in the assessed solutions was performed by HPLC, with respective CT retention times in different solvents (Figure 6).

**Figure 7:**
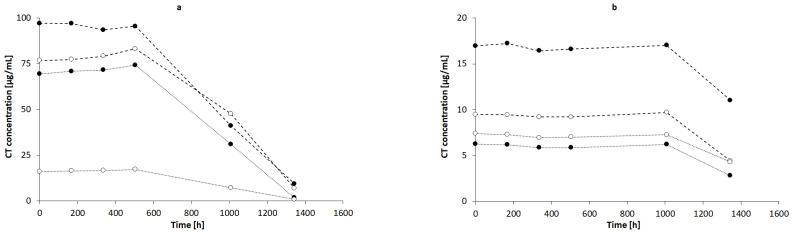

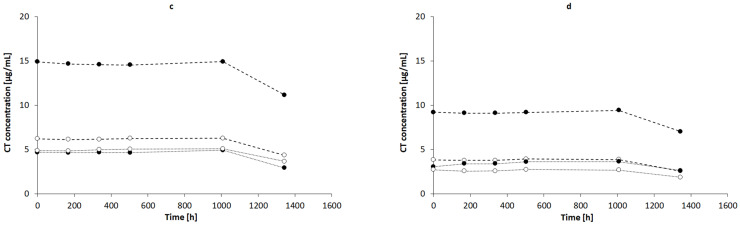
CT concentrations in: (**a**) NaOH solutions of 1 mol/L (-•-), 0.1 mol/L (-◦-), 0.01 mol/L (..•..), and 0.001 mol/L (..◦..) and in 1% alcoholamine solutions of AMPD (-•-), TRIS (-◦-), TEOA (..•..), and TIPA (..◦..) prepared at temperatures of 60 °C (**b**), 50 °C (**c**), and 40 °C (**d**). The lines are guides for the eye.

The highest concentration of CT was observed in NaOH solutions of 1.0, 0.1, and 0.01 mol/L, respectively (Figure 7a), whereas the concentration of CT in 0.001 mol/L NaOH was close to the concentration of CT in 1% AMPD solution (Figure 7b). The CT concentrations in other alcoholamines, TRIS, TEOA, and TIPA, were ca. ten-fold lower than the CT concentrations in NaOH of 1.0, 0.1, and 0.01 mol/L concentrations. An increase in the values of CT concentrations was observed with an increase in the temperature of the solution prepared with alcoholamines (Figure 7b–d). Moreover, a decrease in the concentration of CT in NaOH solutions was noticed after 21 days, whereas in the case of CT in alcoholamine solutions, a decrease was observed after 42 days (Figure 7).

## 4. Discussion

**Figure 8:**
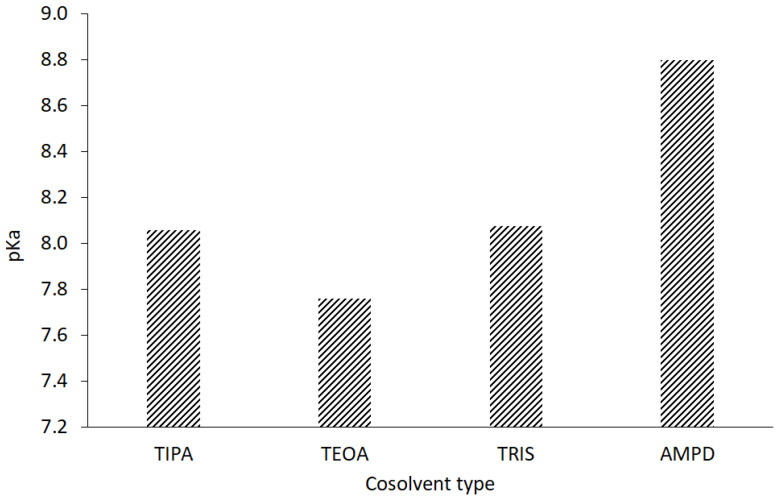
Acidic dissociation constants of the evaluated alcoholamines.

**Figure 9:**
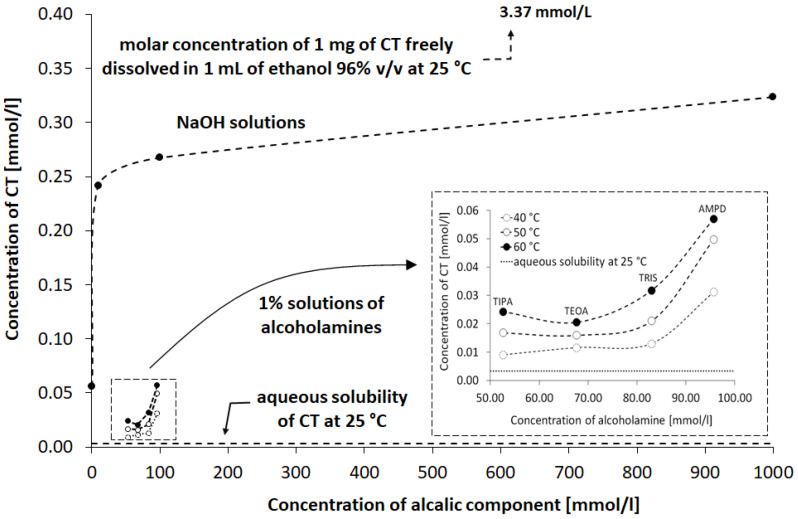
Influence of NaOH solutions and alcoholamine solutions on the observed concentration of CT in aqueous solution compared to the CT solution in water and in ethanol. Details are provided in the text.

The stability of the CT molecule depends on the pH value. Below pH 2, CT tends to be degraded, whereas over pH 11, structural changes are observed [36]. The pH range of 2–8 mildly influences the solubility, whereas pH 10 to 12 strongly favors the aqueous dissolution of CT [4]. In our study, an alkaline environment between pH values of 10.0 and 12.70 was chosen to establish optimal conditions for CT solubility and stability in the solution. Concentrations of 0.001, 0.01, 0.1, and 1.0 mol/L sodium hydroxide were selected with pH values of 10.07, 11.70, 12.55, and 12.66, respectively. The solutions of 1% of aminomethyl propanediol (AMPD), tromethamine (TRIS), triethanolamine (TEOA), and triisopropanolamine (TIPA) were characterized by the following pH values: 10.08, 9.16, 9.46, and 9.18 (Table 2). The relationship between the aqueous CT solubility and pKa of selected solvents was confirmed. The variability of the pKa of alcoholamines (Figure 8) was reflected in the solubility pattern, where the AMPD with the highest pKa provided the best results in terms of CT solubility compared with Figure 9 (inserted panel).

### 4.1. Visual Results

Despite the numerous therapeutic properties of CT, a diterpene compound, its practical application encounters hindrances, resulting from low water solubility and low bioavailability [3]. A visual examination of the impact of the series of solvents on the solubility of CT illustrated high solubility in alcohols, i.e., methanol, ethanol, and isopropanol. Lower solubility was observed in sodium hydroxide solutions, whereas CT was practically insoluble in distilled water, sulfuric acid (VI) solution, hydrochloric acid solution, and alcoholamine solution (Figure 3). In alcoholic solvents and in sodium hydroxide solution, CT dissolved to form yellow-colored solutions, which may be regarded as a confirmation of CT dissolution. Alcohols with a low molecular mass favor the solubility of CT, which was availed in solubility studies [45,46]. However, the presence of long hydrocarbon chains destroys the association between water and alcohol. We observed an increase in the color of the alcoholic solution of CT (Figure 3, left panel). Our observation showed that the addition of water to the solution of CT dissolved in alcohol caused the precipitation of CT, whereas the addition of 0.1 mol/L NaOH solution triggered better CT dissolution. Different dispersions and particle sizes of CT were observed under 10× microscope magnification. There were many differences between the crystal form of CT and that dissolved in ethanol. It was noticed that under the influence of the addition of 0.11 M NaOH solution, the CT particles were broken down and dispersed to a greater extent compared to the sample in pure ethanol. The addition of water resulted in the formation of elongated and thin structures (Figure 3, right panel). The varied behavior of the CT alcoholic solution after the addition of water or NaOH solution should be further examined to elucidate the possible intermolecular interactions, resulting from the slightly acidic nature of alcohols.

### 4.2. The Approximated Concentrations of CT under the Influence of Various Alkaline Solutions

The variability of concentrations obtained from the spectrophotometric assays indicated that the solubility of CT in alkaline solutions was limited, and saturation could be deduced when the volume of the alkaline component was in the range of 4.5–6 mL (Figure 4). The concentration of the alkaline component of ca. 0.05–0.1 mol/L for the alcoholamines resulted in CT dissolution of ca. 15–30 mg/L, whereas the NaOH solution of a much lower concentration of 0.001 mol/L provided comparable solubility of ca. 10 mg/L. The obtained saturation values were of preliminary guidance quality data due to the high volume of added solutions. However, the calculation of the CT/alkaline component ratio provided an assumption to the statement that the CT saturation state in the assessed aqueous systems is possible in the presence of ca. 27 particles of NaOH per one particle of CT, whereas in the case of alcoholamines, the ratio was in the range of ca. 737 to 1514 particles per one particle of CT. The difference may be connected to the lower alkalinity of alcoholamines compared to NaOH; however, the pattern is disturbed by the higher pH in the case of the AMPD solution compared to the NaOH solution (Table 2). Other factors, such as the logP of the alkaline components, may also influence the solubility of CT. The logP may be of special interest for the apparent permeability coefficient in cellular monolayers [3].

### 4.3. Titration Studies

**Figure 10:**
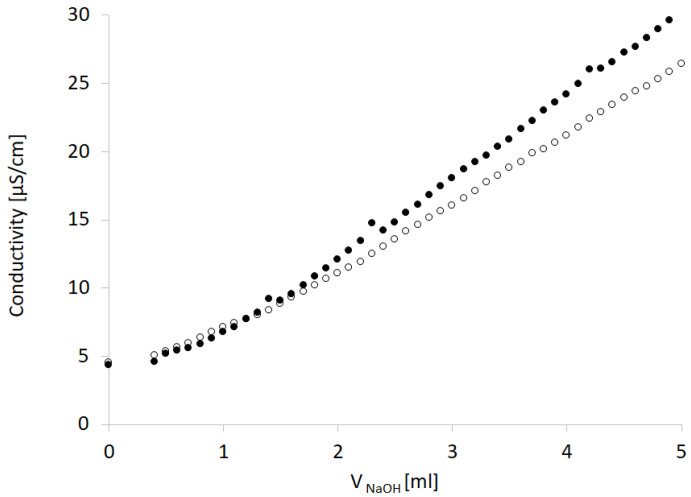
Effect of the addition of 3.3% ethanol (black dots) to the NaOH aqueous solution (empty dots) on the conductivity assessed in the titration procedure.

Evaluation of the influence of increasing concentrations of NaOH on the pH and conductivity of CT dispersions revealed that only slight differences were observed when CT was present in the system. The pH plot for the titration of CT was adjacent to the plot for the titration of water (Figure 5a). The course of the dependence was compared to the case of the neutralization of CO_2_, usually present in water; however, we used deionized water. The conductivity data provided more insight into the CT–water–NaOH interaction (Figure 5b). In the presence of CT, the conductivity increased faster compared to the aqueous system. This increase was effectively observed after CO_2_ neutralization, which was confirmed by plotting the pH with the experimental and theoretical curves. CO_2_ neutralization took place at approximately 1 mL of NaOH 0.001 mol/L. The higher conductivity values (black dots) reflected the dissolution of CT under the influence of NaOH solution. Interestingly, when the ethanol–water system was applied in the same conditions, the pH variability between the sample with CT and without CT was less pronounced, supposedly due to the lower content of ionizable water generating the protons (Figure 5c). The conductivity pattern suggests that ethanol supported the dissolution of CT, which is visible when comparing the plots in Figure 5b,d. The conductivity at the initial point was higher when the ethanol was present in the sample, and also, an increasing conductivity tendency was observed. Ethanol has low conductivity compared to water [47,48], and the presence of alcohol should favor a decrease in conductivity; however, in the presence of NaOH, a reduction in conductivity was not noticed (Figure 10).

### 4.4. HPLC Studies

The HPLC method was widely applied to qualitatively and quantitatively assay CT in several compositions [5,49], as well as the active substances of *Salvia miltiorrhiza* [27] or the CT release processes [32]. Our HPLC studies confirmed the correlation between the temperature conditions of the dissolving procedure. The increased temperature favored the higher solubility of CT in the solutions of alcoholamines (Figure 10, inserted plot). The NaOH solutions enabled an increase in CT concentrations up to the range of 0.056–0.320 mmol/L, depending on the NaOH concentrations. When compared to NaOH solutions, the 1% solutions of TRIS, TEOA, and TIPA provided a moderate increase in CT aqueous solubility. The resulting CT concentrations were in the range of 0.01–0.03 mmol/L compared to the aqueous solubility of CT of ca. 0.003 mmol/L. The 1% AMPD solution was distinguished out of the alcoholamine solutions and enabled the highest concentrations of CT in the range of 0.03–0.06 mol/L, and CT solubilization varied depending on the temperature of the dissolution process (Figure 9).

**Figure 11:**
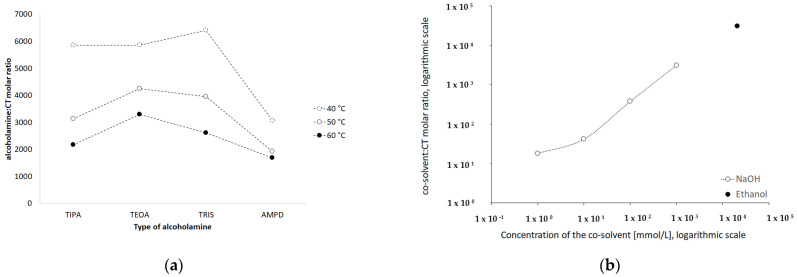
Influence of type of the alcoholamine on the molar ratio alcoholamine/CT in solutions prepared in varied temperature (**a**) and the influence of increasing concentrations of NaOH and of 96% ethanol on the molar ratio cosolvent/CT (**b**).

The molar ratios of alcoholamine/CT achieved the lowest values for the AMPD solution, which confirms its highest efficacy in terms of the CT solubility increase in aqueous solutions of the selected alcoholamines—AMPD, TRIS, TEOA, and TIPA (Figure 11a). The stability of CT was higher in the solutions of alcoholamines compared to the NaOH solutions, which is very interesting from the practical point of view. The stable content of CT was retained two-fold longer in the case of alcoholamines, i.e., for 42 days, whereas the NaOH solutions retained the initial level of CT only for ca. 21 days.

The molecular peculiarity of AMPD in comparison to TEOA, TIPA and TRIS is based on the number of OH groups in the particle, which are attached to the nonlinear carbon chain with the primary amine group. Both the OH groups’ number and the number of carbon chains attached to the nitrogen atom may influence the dissolving potential of alcoholamines against CT (Figure 10). The number of NaOH molecules interacting with CT increases with the increase in NaOH concentration, and the pattern is close to the linear dependence. The ethanol solution fits the pattern observed for NaOH solutions (Figure 11b). The stability of CT solutions in alkaline solutions depended on the type of alkaline component. In the NaOH solutions, regardless of the NaOH concentrations, the level of CT decreased after 21 days, whereas in the case of alcoholamines, the CT level decreased after 42 days (Figure 7a–d). The short shelf time of CT may depend on the storage conditions of the solutions at 25 °C in the absence of direct light. CT solutions were also heated up to 40–60 °C, as temperature may affect the solubility of CT. CT is temperature sensitive, so the producer’s specification recommends storage of the powder at –20 °C and CT in solvent at −80 °C [50]. Song and coworkers found the instability of CT in methanol at room temperature and after exposure to light [51]. Our research was preparatory to the final hydrogel’s formulation of CT. Further steps will include stabilization of the samples via proper storage of the mixtures, e.g., in low temperatures, in the absence of direct light, or via inhibition of the degradation processes with the use of selected substances, which will be developed in the future. However, the most interesting option includes the ex tempore preparation of the mixtures prescribed for an individual patient. Some preparations against acne have been developed in the ex tempore form, i.e., due to the instability of erythromycin in suspension with zinc salt, clindamycin hydrochloride in lotion [52], or azelaic acid in a liposomal formulation [53].

## 5. Conclusions

Alcohols, including methanol, ethanol, and isopropanol, are good solvents for CT. The alkalic environment of NaOH solutions, as well as the alcoholamine solutions, favor the dissolution of CT. CT dissolution is mainly dependent on the pH, and the presence of ethanol is a conducive factor for CT aqueous solubility, similar to the increased temperature and sonification applied during the dissolution procedure. There are only slight differences between TIPA, TEOA, and TRIS in the solubilizing potential. Between the analyzed alcoholamines, AMPD seems to be the most interesting alkalic component, which may be used to enhance the solubility of CT, as confirmed by the number of AMPD particles on the CT particle in the aqueous solution. Alcoholamines enable higher stability compared to NaOH in respective solutions, which is very important for the further development of CT in various pharmaceutical formulations.

## Figures and Tables

**Table 1:** Color appearance in the 5 mg CT sample in the 0.1 mol/L NaOH solution under the influence of ultrasounds and increased temperature for 1–10 min. P—presence of color; A—absence of color.

Time (min)	1	5	10
Temperature (°C)			
30	A	A	P
40	A	P	P
50	P	P	P
60	P	P	P

**Table 2:** The results of the pilot study of the solubility of 5 mg of CT, i.e., 1.68·10^−5^ mol, in the alkaline solutions of NaOH, AMPD, TRIS, TEOA, and TIPA.

Solvent	Solution of:				
	NaOH	AMPD	TRIS	TEOA	TIPA
Molar mass (g/mol)	39.99	105.14	121.14	149.18	191.27
Alkalic component concentration (mol/L)	0.001	0.095	0.083	0.067	0.052
The plateau volume (mL)	6.0	5.0	4.5	5.0	4.5
Amount of alkali component (mmol)	0.0066	0.475	0.374	0.335	0.234
The preliminary molar ratio alkali component/CT	27	889	1514	1089	737
pH after establishing the plateau volume (8 mL)	10.07 ± 0.20	10.08 ± 0.10	9.16 ± 0.20	9.46 ± 0.10	9.18 ± 0.20

**Table 3:** Equations of standard plots for the evaluation of CT concentration in the HPLC assays.

Standard Solution	Retention Time (min)	Slope	Intercept	r^2^	Linearity (µg/mL)	Peak Area (mAU × min)
S1	17.6361	0.4839	0.0948	0.999	1–15	0.5–6
S2	17.6361	0.4302	0.8644	0.999	20–235	10–105

## Data Availability

Not applicable.

## Supplementary Materials

The following are available online at https://www.mdpi.com/article/10.3390/pharmaceutics13070992/s1, Table S1: Factors applied for dissolution of 1 mg of CT, evaluated in the terms of visual appearance, Table S2: The composition of samples applied in the titration studies, Table S3: The composition and preparation parameters of the HPLC samples.

## References

[1] Vemula V.R., Lagishetty V., Lingala S. (2010). Solubility enhancement techniques. Int. J. Pharmac. Sci. Rev. Res..

[2] Kavitha K., Vishva Ranjani P. (2015). Solubility enhancement—A challenge for hydrophobic drugs. IOSR J. Pharm. Biol. Sci..

[3] Zhang J., Huang M., Guan S., Bi H.-C., Pan Y., Duan W., Chan S.Y., Chen X., Hong Y.-H., Bian J.-S. (2006). A mechanistic study of the intestinal absorption of cryptotanshinone, the major active constituent of Salvia miltiorrhiza. J. Pharmacol. Exp. Ther..

[4] Chen W., Lu Y., Chen G., Huang S. (2013). Molecular Evidence of Cryptotanshinone for Treatment and Prevention of Human Cancer. Anticancer Agents Med. Chem..

[5] Xing L., Tan Z.-R., Cheng J.-L., Huang W.-H., Zhang W., Deng W., Yuan C.-S., Zhou H.-H. (2017). Bioavailability and pharmacokinetic comparison of tanshinones between two formulations of Salvia miltiorrhiza in healthy volunteers. Sci. Rep..

[6] Jiang Y.-Y., Li Q., Lu W., Cai J.-C. (2003). Facile and efficient total synthesis of (±)-cryptotanshinone and tanshinone IIA. Tetrahedron Lett..

[7] Zhou L., Zuo Z., Chow M.S.S. (2005). Danshen: An Overview of Its Chemistry, Pharmacology, Pharmacokinetics, and Clinical Use. J. Clin. Pharmacol..

[8] Li J., Bai Y., Bai Y., Zhu R., Liu W., Cao J., Yan-Xu C., Tan Z., Chang Y.-X. (2017). Pharmacokinetics of Caffeic Acid, Ferulic Acid, Formononetin, Cryptotanshinone, and Tanshinone IIA after Oral Administration of Naoxintong Capsule in Rat by HPLC-MS/MS. Evid. Based Complement Altern. Med..

[9] Liu Z., Xu S., Huang X., Wang J., Gao S., Li H., Zhou C., Ye J., Chen S., Jin Z.-G. (2015). Cryptotanshinone, an orally bioactive herbal compound from Danshen, attenuates atherosclerosis in apolipoprotein E-deficient mice: Role of lectin-like oxidized LDL receptor-1 (LOX-1). Br. J. Pharmacol..

[10] Zhang Y., Won S.-H., Jiang C., Lee H.-J., Jeong S.-J., Lee E.-O., Zhang J., Ye M., Kim S.-H., Lu J. (2012). Tanshinones from Chinese Medicinal Herb Danshen (Salvia miltiorrhiza Bunge) Suppress Prostate Cancer Growth and Androgen Receptor Signaling. Pharm. Res..

[11] Krajewska-Patan A., Dreger M., Górska-Paukszta M., Mścisz A., Mielcarek S., Baraniak M., Buchwald W., Marecik R., Grajek W., Mrozikiewicz P.M. (2007). Salvia milthiorrhiza Bunge in vitro cultivation in callus cultures. Herba Pol..

[12] Hügel H.M., Jackson N. (2014). Danshen diversity defeating dementia. Bioorg. Med. Chem. Lett..

[13] Rimmon A., Vexler A., Berkovich L., Earon G., Ron I., Lev-Ari S. (2013). Escin Chemosensitizes Human Pancreatic Cancer Cells and Inhibits the Nuclear Factor-kappaB Signaling Pathway. Biochem. Res. Int..

[14] Guan S., Ma J., Zhang Y., Gao Y., Zhang Y., Zhang X., Wang N., Xie Y., Wang J., Zhang J. (2013). Danshen (Salvia miltiorrhiza) injection suppresses kidney injury induced by iron overload in mice. PLoS ONE.

[15] Xing S., Zhao L., Cao Q. (2017). Safety Problem of Gynecological Disease Treatment Herb Danshen. Lat. Am. J. Pharm..

[16] Ren-An Q., Juan L., Chuyuan L., Wenjuan F., Chunyan H., Xuemei Y., Lin H., Hong N. (2014). Study of the protective mechanisms of Compound Danshen Tablet (Fufang Danshen Pian) against myocardial ischemia/reperfusion injury via the Akt-eNOS signaling pathway in rats. J. Ethnopharmacol..

[17] Jin H.-J., Li C.-G. (2016). Molecular Mechanisms of Cardioprotective Actions of Tanshinones. J. Chem..

[18] Jin Y.C., Kim C.W., Kim Y.M., Nizamutdinova I.T., Ha Y.M., Kim H.J., Seo H.G., Son K.H., Jeon S.J., Kang S.S. (2009). Cryptotanshinone, a lipophilic compound of Salvia miltiorrriza root, inhibits TNF-α-induced expression of adhesion molecules in HUVEC and attenuates rat myocardial ischemia/reperfusion injury in vivo. Eur. J. Pharmacol..

[19] Ahmad Z., Ng C.T., Fong L.Y., Abu Bakar N.A., Hussain N.H.M., Ang K.P., Ee G.C.L., Hakim M.N. (2016). Cryptotanshinone inhibits TNF-α-induced early atherogenic events in vitro. J. Physiol. Sci..

[20] Maione F., Piccolo M., De Vita S., Chini M.G., Cristiano C., De Caro C., Lippiello P., Miniaci M.C., Santamaria R., Irace C. (2018). Down regulation of pro-inflammatory pathways by tanshinone IIA and cryptotanshinone in a non-genetic mouse model of Alzheimer’s disease. Pharmacol. Res..

[21] Shen D.-Y., Kang J.-H., Song W., Zhang W.-Q., Li W.-G., Zhao Y., Chen Q.-X. (2011). Apoptosis of Human Cholangiocarcinoma Cell Lines induced by β-Escin through Mitochondrial Caspase-dependent Pathway. Phytother. Res..

[22] Ling W., Xuehua J., Weijuan X., Chenrui L. (2007). Complexation of tanshinone IIA with 2-hydroxypropyl-beta-cyclodextrin: Effect on aqueous solubility, dissolution rate, and intestinal absorption behavior in rats. Int. J. Pharm..

[23] Chen Z., Zhu R., Zheng J., Chen C., Huang C., Ma J., Xu C., Zhai W., Zheng J. (2017). Cryptotanshinone inhibits proliferation yet induces apoptosis by suppressing STAT3 signals in renal cell carcinoma. Oncotarget.

[24] Cha J.-D., Jeong M.-R., Choi K.-M., Park J.-H., Cha S.-M., Lee K.-Y. (2013). Synergistic effect between cryptotanshinone and antibiotics in oral pathogenic bacteria. Adv. Biosci. Biotechnol..

[25] Lee D.-S., Lee S.-H., Noh J.-G., Hong S.-D. (1999). Antibacterial Activities of Cryptotanshinone and Dihydrotanshinone I from a Medicinal Herb, Salvia miltiorrhiza Bunge. Biosci. Biotechnol. Biochem..

[26] Wang D., Zhang W., Wang T., Li N., Mu H., Zhang J., Duan J. (2015). Unveiling the mode of action of two antibacterial tanshinone derivatives. Int. J. Mol. Sci..

[27] Yu H., Subedi R.K., Nepal P.R., Kim Y.G., Choi H.K. (2012). Enhancement of solubility and dissolution rate of cryptotanshinone, tanshinone i and tanshinone IIA extracted from Salvia miltiorrhiza. Arch. Pharm. Res..

[28] Kakisawa H., Inouye Y. (1968). Total Syntheses of Tanshinone-I, Tanshinone-II and Cryptotanshinone. Chem. Commun..

[29] Zhai X., Li C., Lenon G.B., Xue C.C.L., Li W. (2017). Preparation and characterisation of solid dispersions of tanshinone IIA, cryptotanshinone and total tanshinones. Asian J. Pharm. Sci..

[30] Hu L., Xing Q., Meng J., Shang C. (2010). Preparation and enhanced oral bioavailability of cryptotanshinone-loaded solid lipid nanoparticles. AAPS PharmSciTech.

[31] Yu Z., Lv H., Han G., Ma K. (2016). Ethosomes Loaded with Cryptotanshinone for Acne Treatment through Topical Gel Formulation. PLoS ONE.

[32] Wang Z., Liu L., Xiang S., Jiang C., Wu W., Ruan S., Du Q., Chen T., Xue Y., Chen H. (2020). Formulation and Characterization of a 3D-Printed Cryptotanshinone-Loaded Niosomal Hydrogel for Topical Therapy of Acne. AAPS PharmSciTech.

[33] Zuo T., Chen H., Xiang S., Hong J., Cao S., Weng L., Zhang L., Liu L., Li H., Zhu H. (2016). Cryptotanshinone-Loaded Cerasomes Formulation: In Vitro Drug Release, In Vivo Pharmacokinetics, and In Vivo Efficacy for Topical Therapy of Acne. ACS Omega.

[34] Li J.F., Wei Y.X., Ding L.H., Dong C. (2003). Study on the inclusion complexes of cryptotanshinone with β-cyclodextrin and hydroxypropyl-β-cyclodextrin. Spectrochim. Acta Part A.

[35] Dong C., Qiao J., Yang P. (2000). Study on the fluorescence properties of cryptotanshinone and tanshinone IIA and their molecular structures in different acidic media. Chin. J. Spectrosc. Lab..

[36] Li J.F., Wei Y.X., Xu Z.C., Dong C., Shuang S.M. (2004). Studies on the spectroscopic behavior of cryptotanshinone, tanshinone IIA, and tanshinone I. Spectrochim. Acta Part A.

[37] Fiume M.M., Heldreth B., Bergfeld W.F., Belsito D.V., Hill R.A., Klaassen C.D., Liebler D., Marks J.G., Shank R.C., Slaga T.J. (2013). Safety Assessment of Triethanolamine and Triethanolamine-Containing Ingredients as Used in Cosmetics. Int. J. Toxicol..

[38] Musial W., Kubis A. (2006). Preliminary evaluation of interactions between selected alcoholamines and model skin sebum components. Chem. Pharm. Bull..

[39] Kostrzębska A., Musiał W. (2020). The influence of increasing concentrations of AMPD on the efficacy of its penetration into a model skin sebum layer. Pharmaceutics.

[40] Burnett C.L., Bergfeld W.F., Belsito D.V., Klaassen C.D., Marks J.G., Shank R.C., Slaga T.J., Snyder P.W., Andersen F.A. (2009). Final amended report on safety assessment on aminomethyl Propanol and aminomethyl propanediol. Int. J. Toxicol..

[41] Liebert M.A. (1987). Final Report on the Safety Assessment of Diisopropanolamine, Triisopropanolamine, Isopropanolamine, and Mixed Isopropanolamine. J. Am. Coll. Toxicol..

[42] Becker L.C., Bergfeld W.F., Belsito D.V., Hill R.A., Klaassen C.D., Liebler D.C., Marks J.G., Shank R.C., Slaga T.J., Snyder P.W. (2018). Safety Assessment of Tromethamine, Aminomethyl Propanediol, and Aminoethyl Propanediol as Used in Cosmetics. Int. J. Toxicol..

[43] Shi Z., He J., Yao T., Chang W., Zhao M. (2005). Simultaneous determination of cryptotanshinone, tanshinone I and tanshinone IIA in traditional Chinese medicinal preparations containing Radix salvia miltiorrhiza by HPLC. J. Pharm. Biomed. Anal..

[44] (2017). Polish Pharmacopeia XI.

[45] Song M., Hang T.J., Zhang Z., Chen H.Y. (2007). Effects of the coexisting diterpenoid tanshinones on the pharmacokinetics of cryptotanshinone and tanshinone IIA in rat. Eur. J. Pharm. Sci..

[46] Kim H.-K., Woo E.-R., Lee H.-W., Park H.-R., Kim H.-N., Jung Y.-K., Choi J.-Y., Chae S.-W., Kim H.-R., Chae H.-J. (2008). The Correlation of *Salvia miltiorrhiza* Extract–Induced Regulation of Osteoclastogenesis with the Amount of Components Tanshinone I, Tanshinone IIA, Cryptotanshinone, and Dihydrotanshinone. Immunopharmacol. Immunotoxicol..

[47] Frank F., Ives D.J.G. (1966). The structural properties of alcohol–water mixtures. Q. Rev. Chem. Soc..

[48] Personna Y., Slater L., Ntarlagiannis D., Werkema D., Szabo Z. (2013). Electrical signatures of ethanol-liquid mixtures: Implications for monitoring biofuels migration in the subsurface. J. Contam. Hydrol..

[49] Cheng H.T., Li X.L., Li X.R., Li Y.H., Wang L.J., Xue M. (2012). Simultaneous quantification of selected compounds from Salvia herbs by HPLC method and their application. Food Chem..

[50] Cryptotanshinone. https://www.selleckchem.com/products/Cryptotanshinone.html.

[51] Song M., Hang T.J., Zhang Z.X., Du R., Chen J. (2005). Determination of cryptotanshinone and its metabolite in rat plasma by liquid chromatography-tandem mass spectrometry. J. Chromat. B.

[52] Dedić M., Bečić E., Imamović B., Žiga N. (2018). Determination of clindamycin hydrochloride content in 1% clindamycin lotion. Bull. Chem. Technol. Bosnia Herzeg..

[53] Han S., Karłowicz-Bodalska K., Potaczek P., Wójcik A., Ozimek Ł., Szura D., Musiał W. (2014). Identification of unknown impurity of azelaic acid in liposomal formulation assessed by HPLC-ELSD, GC-FID, and GC-MS. AAPS PharmSciTech.

