# Supplementary material for: The Influence of Selected Factors on the Aqueous Cryptotanshinone Solubility
Source: Pharmaceutics. 2021 Jun 30;13(7):992. doi: 10.3390/pharmaceutics13070992 (PMC8309180; doi:10.3390/pharmaceutics13070992)
Supplement: Supplementary file 1 [file pharmaceutics-13-00992-s001.zip › pharmaceutics-1264543-supplementary.pdf]

pharmaceutics

Supplementary Material: The Influence of Selected Factors on the Aqueous Cryptotanshinone Solubility

Justyna Kobryń, Jowita Kałużna and Witold Musiał

Table S1. Factors applied for dissolution of 1 mg of CT, evaluated in the terms of visual appearance.

No of Sample	Aqua [mL]	NaOH 0.1 mol/L [mL]	H_2SO_4 96% [mL]	HCl 35–38% [mL]	Me-OH ≥99.9% [mL]	Et-OH 96% [mL]	Is-OH ≥99.5% [mL]	AMP 1% [mL]	TRIS 1% [mL]	TEOA 1% [mL]	TIPA 1% [mL]
1	2.0										
2		2.0									
3			2.0								
4				2.0							
5					2.0						
6						2.0					
7							2.0				
8								2.0			
9									2.0		
10										2.0	
11											2.0

Table S2. The composition of samples applied in the titration studies.

Sample	CT [mg]	Water [mL]	Ethanol [mL]	Titrant Conc. [mol/L]
P1	5.0	36.2	-	0.001
P1r	-	36.2	-	
P2	5.0	35.0	1.2	
P2r	-	35.0	1.2	

Table S3. The composition and preparation parameters of the HPLC samples.

Sample	CT [mg]	NaOH [mol/L]	AMPD [%]	TRIS [%]	TEOA [%]	TIPA [%]	EtOH [g]	Temp. [°C]	Mass [g]	pH
NaOH 0.001 mol/L	0.88	0.001	-	-	-	-	0.42	25	5.27	10.40 ± 0.10
NaOH 0.01 mol/L	0.88	0.010	-	-	-	-	0.42	25	5.22	11.60 ± 0.10
NaOH 0.1 mol/L	0.85	0.100	-	-	-	-	0.42	25	5.05	12.55 ± 0.10
NaOH 1 mol/L	0.86	1.000	-	-	-	-	0.42	25	5.13	12.73 ± 0.20
1% AMPD 40 °C	0.88	-	1	-	-	-	0.42	40	5.04	9.99 ± 0.00
1% AMPD 50 °C	0.81	-	1	-	-	-	0.42	50	5.00	10.11 ± 0.10
1% AMPD 60 °C	0.87	-	1	-	-	-	0.42	60	5.01	10.24 ± 0.00
1% TRIS 40 °C	0.78	-	-	1	-	-	0.42	40	5.03	9.50 ± 0.00
1% TRIS 50 °C	0.88	-	-	1	-	-	0.42	50	5.49	9.65 ± 0.10
1% TRIS 60 °C	0.87	-	-	1	-	-	0.42	60	5.25	9.76 ± 0.01
1% TEOA 40 °C	0.79	-	-	-	1	-	0.42	40	5.06	9.31 ± 0.10
1% TEOA 50 °C	0.87	-	-	-	1	-	0.42	50	5.26	9.32 ± 0.00
1% TEOA 60 °C	0.72	-	-	-	1	-	0.42	60	5.11	9.27 ± 0.01
1% TIPA 40 °C	0.77	-	-	-	-	1	0.42	40	5.00	9.26 ± 0.00
1% TIPA 50 °C	0.87	-	-	-	-	1	0.42	50	5.10	9.48 ± 0.20
1% TIPA 60 °C	0.88	-	-	-	-	1	0.42	60	5.01	9.51 ± 0.01
Ethanol 96%	1.10	-	-	-	-	-	5.01	25	5.01	7.30±0.10
Water40 °C	1.00	-	-	-	-	-	-	40	5.00	6.54 ± 0.10
Water50 °C	1.10	-	-	-	-	-	-	50	5.03	6.95 ± 0.10
Water 60 °C	1.10	-	-	-	-	-	-	60	5.02	6.97 ± 0.10
